# All-fibre photonic signal generator for attosecond timing and ultralow-noise microwave

**DOI:** 10.1038/srep16250

**Published:** 2015-11-04

**Authors:** Kwangyun Jung, Jungwon Kim

**Affiliations:** 1 School of Mechanical and Aerospace Engineering, Korea Advanced Institute of Science and Technology (KAIST), Daejeon 305-701, Korea

## Abstract

High-impact frequency comb applications that are critically dependent on precise pulse timing (i.e., repetition rate) have recently emerged and include the synchronization of X-ray free-electron lasers, photonic analogue-to-digital conversion and photonic radar systems. These applications have used attosecond-level timing jitter of free-running mode-locked lasers on a fast time scale within ~100 μs. Maintaining attosecond-level absolute jitter over a significantly longer time scale can dramatically improve many high-precision comb applications. To date, ultrahigh quality-factor (Q) optical resonators have been used to achieve the highest-level repetition-rate stabilization of mode-locked lasers. However, ultrahigh-Q optical-resonator-based methods are often fragile, alignment sensitive and complex, which limits their widespread use. Here we demonstrate a fibre-delay line-based repetition-rate stabilization method that enables the all-fibre photonic generation of optical pulse trains with 980-as (20-fs) absolute r.m.s. timing jitter accumulated over 0.01 s (1 s). This simple approach is based on standard off-the-shelf fibre components and can therefore be readily used in various comb applications that require ultra-stable microwave frequency and attosecond optical timing.

Recent advances in ultralow-noise mode-locked lasers and optical frequency combs have enabled new applications that directly and critically depend on precise pulse timing (i.e., repetition rate); some compelling examples include ultralow-phase-noise microwave and millimetre-wave synthesis^1^,^2^, photonic analogue-to-digital conversion^3^,^4^, timing synchronization for X-ray free-electron lasers^5^,^6^ and ultrafast electron diffraction^7^, time-of-flight-based sensing and ranging^8^,^9^, comb-based remote time-frequency transfer^10^, photonic radar systems^11^, and on-chip clock distribution networks^12^. Many of these applications have been enabled by the attosecond-level timing jitter of free-running solid-state or fibre mode-locked lasers on a fast time scale (e.g., timing jitter accumulated over a time scale of <100 μs; integrated for >10 kHz Fourier frequency)^13^,^14^,^15^. However, the jitter of free-running lasers rapidly diverges over longer time scales; a recent study showed that the absolute r.m.s. jitter of free-running fibre lasers reaches 20 ps over 1 s despite exhibiting only 710-as jitter within 100 μs^16^. Therefore, maintaining attosecond-level absolute timing jitter over much longer time scales (e.g., 10 ms or longer) could significantly benefit many existing applications and also open up new applications in precision timing both in optics and electronics.

To suppress the repetition-rate phase noise in lower Fourier frequency, free-running mode-locked lasers can be locked to an ultrahigh quality-factor (Q) radio frequency (rf), microwave or optical reference. Locking to state-of-the-art room-temperature rf/microwave sources is insufficient to achieve sub-100-fs absolute timing jitter at a time scale of ~1 s. For the highest level of performance, optical references such as ultrahigh-Q (i.e., ~10^11^) Fabry-Perot cavities^1^,^17^ or micro-resonators^18^ have been used. To date, in most cases, narrow-linewidth continuous-wave (cw) lasers have been used as connecting links between optical resonators and mode-locked lasers. However, the use of cw lasers requires multi-stage phase-locked loops (PLLs) for both the repetition rate (*f*_rep_) and the carrier-envelope offset frequency (*f*_ceo_), which involves non-trivial tasks such as octave-spanning spectral broadening and *f*_ceo_ detection (and control) via *f*–2*f* interference. Ultrahigh-Q optical resonators and their shielding systems are also typically fragile and alignment-sensitive, and require high-quality vacuum systems and vibration-isolation platforms. Therefore, a simpler and more robust repetition-rate stabilization method that can still achieve both ultralow phase noise/timing jitter and ultrahigh repetition-rate frequency stability is highly desirable.

Here we demonstrate a new all-fibre-based method for the repetition-rate stabilization of mode-locked lasers without using any cw lasers, spectral broadening or *f*_ceo_ detection. This novel method is based on the direct repetition-rate stabilization of a mode-locked laser to a kilometre-scale fibre delay line. Using this method to stabilize a mode-locked Er-fibre laser enables the all-fibre photonic generation of optical pulse trains with sub-femtosecond absolute timing jitter over a 0.01-s time scale, which is ~100 times longer than free-running lasers. Over a 1-s time scale, the integrated absolute r.m.s. timing jitter is suppressed by a factor of 500 from 10 ps to only 20 fs.

## Results

### Operating principle

**Figure 1:**
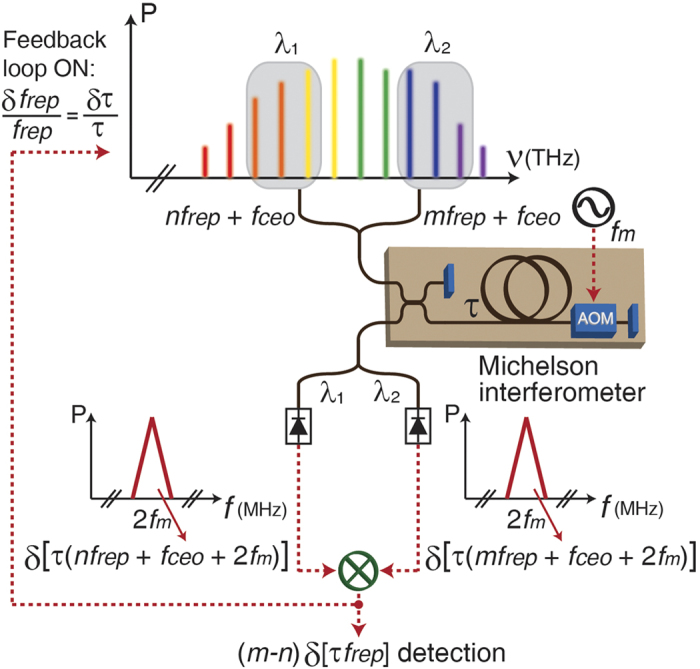
Working mechanism of the frequency comb repetition-rate stabilization to the fibre-delay line. Two spectral regions in the frequency comb are applied to an all-fibre Michelson interferometer with a time delay of τ Via synchronous detection, the repetition-rate frequency noise at (*m*–*n*)*f*_rep_ is detected and further used to lock the repetition-rate of a frequency comb to the fibre-delay line. AOM, acousto-optic modulator.

The concept of repetition-rate stabilization is based on optical interference by an all-fibre Michelson interferometer that includes an optical-fibre delay (Fig. 1). Unlike previous studies that used this optical-fibre delay to suppress frequency noise in cw lasers^19^,^20^,^21^,^22^, this study uses the optical-fibre delay to directly stabilize the repetition-rate of a mode-locked laser using two separate spectral regions in the frequency comb. Using the optical carrier interference between a reference arm and a long delay arm, the absolute frequency noise of comb modes (*nf*_rep_* + f*_ceo_) is detected. To extract only the repetition-rate noise, the carrier-envelope-offset frequency (*f*_ceo_) noise should be eliminated; this can be achieved by common-mode *f*_ceo_ rejection using two spectral regions (i.e., λ_1_ and λ_2_ in Fig. 1) of the frequency comb^23^,^24^,^25^. Synchronous detection using an acousto-optic modulator (AOM), which is driven by the rf-frequency *f*_*m*_ in the delay arm, is further used to suppress the background noise during phase detection. At the interferometer output, each wavelength component (i.e., λ_1_ and λ_2_) is split and detected by photodetectors. Each photodetected power contains the frequency noise of the corresponding frequency modes (i.e., (*nf*_*rep*_* + f*_*ceo*_* + *2*f*_*m*_) for λ_1_ and (*mf*_*rep*_* + f*_*ceo*_* + *2*f*_*m*_) for λ_2_) weighted by the delay time (τ) in the form of phase noise at 2*f*_*m*_ (i.e., twice the AOM driving frequency). The common frequency components (*f*_*ceo*_* + *2*f*_*m*_) are rejected by a frequency mixer, and only the repetition-rate frequency noise, δ(*m*–*n)f*_rep_, is detected as a baseband error signal, which can be used for feedback control. When the comb repetition rate is locked to the fibre delay, the fractional frequency instability (δ*f*_rep_/*f*_rep_) follows the fractional delay time of the long fibre link (δτ/τ). To improve the phase detection sensitivity and therefore the achievable stabilization performance, the wavelength spacing (λ_1_–λ_2_), delay time (τ), fibre scattering-limited relative intensity noise (RIN), and power-dependent RIN should be all considered and optimized (see Supplementary Information for detailed discussion on the phase noise scalability).

### Experimental implementation

**Figure 2:**
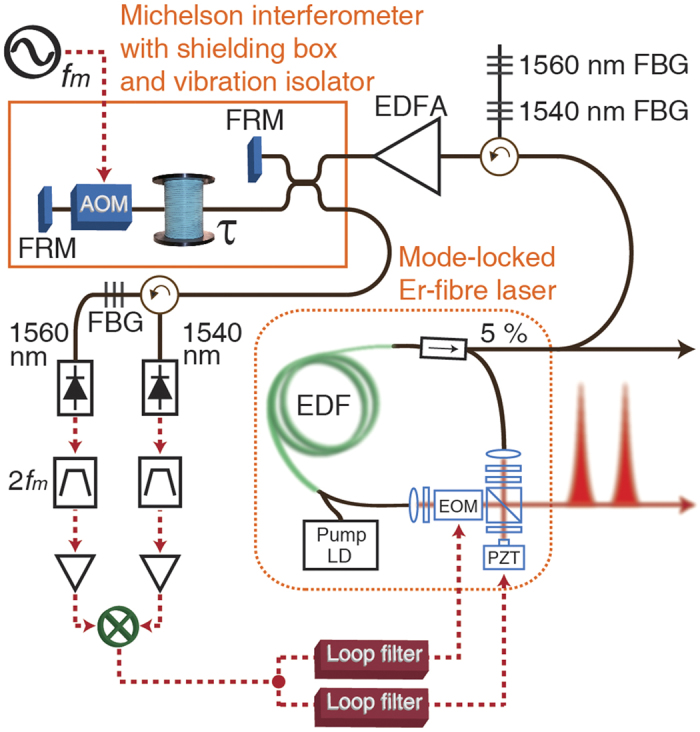
All-fibre photonics-based, ultralow-jitter optical pulse train source using a mode-locked fibre laser and a fibre-delay line-based stabilization method. The repetition-rate frequency noise of the 77-MHz stretched-pulse mode-locked Er-fibre laser is locked to a 2.5-km-long fibre-delay line in the Michelson interferometer. AOM, acousto-optic modulator; EDFA, Er-doped fibre amplifier; EOM, electro-optic phase modulator; FBG, fibre Bragg grating; FRM, Faraday rotating mirror; PZT, piezoelectric transducer.

Figure 2 shows the experimental setup that demonstrates a fibre-photonics-based, ultralow-jitter optical pulse train source using a mode-locked fibre laser and the proposed fibre-delay line-based stabilization method. A dispersion-managed soliton mode-locked Er-fibre laser with a 77-MHz repetition rate and an 840-attosecond high-frequency timing jitter, which is integrated from 10-kHz to 10-MHz Fourier frequency, is used as the frequency comb source. Two wavelength components of the output frequency comb (i.e., at 1540 and 1560 nm with a 1-nm bandwidth) that are filtered by fibre Bragg gratings (FBGs) are amplified by an Er-doped fibre amplifier (EDFA) and applied to the Michelson interferometer. A spool of 1.25-km-long, dispersion-compensated fibre with an in-line AOM modulated at 50 MHz is used as the delay arm. Faraday rotating mirrors (FRMs) are used as the end mirrors of both the reference and delay arms of the interferometer to ensure polarization-independent interference. To suppress the influence of acoustic noise, vibration and air fluctuations, the fibre interferometer with a footprint of 30 cm by 30 cm is placed in a shielding box on a passive vibration isolation platform (see Supplementary Information). After photodetection, the obtained 100-MHz rf-signals (2*f*_*m*_) are filtered by rf-bandpass filters, amplified, and then frequency-mixed. After the mixer output undergoes low-pass filtering, the baseband error signal is obtained. The detected error signal is then split and applied to a piezoelectric transducer (PZT)-mounted mirror and an electro-optic phase modulator (EOM) inside the mode-locked laser through loop filters for slow and fast feedbacks, respectively.

For the out-of-loop measurement of the absolute phase noise in the repetition rate, two different methods are used in a complementary way. First, we built another fibre-delay line-based interferometer setup and used it as the out-of-loop phase detector^22^. Second, we used the well-established balanced optical cross-correlation (BOC) method^16^. The reason why we used the BOC method is two-fold: 1) it is used to crosscheck the validity of the fibre-delay method when measuring the phase noise; and 2) it is used to accurately measure the phase noise and timing jitter spectra outside of the 1/τ frequency, where the detection sensitivity and accuracy of the fibre-delay method is limited compared to the BOC method. More information on the measurement methods used in this study is provided in Methods and Supplementary Information.

### Measurement results

**Figure 3:**
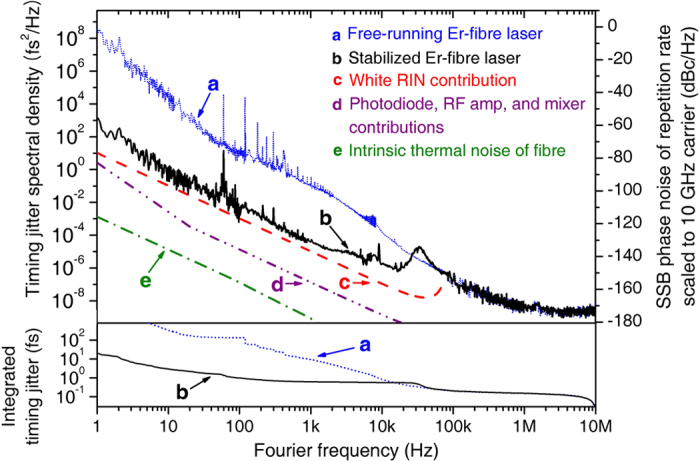
Power spectral densities of absolute timing jitter and repetition-rate phase noise (scaled to a 10-GHz carrier frequency) of the mode-locked Er-fibre laser. (**a**) Free-running Er-fibre laser. (**b**) Stabilized Er-fibre laser. (**c**) Projected phase noise by the relative intensity noise (RIN) from the Rayleigh scattering in the 2.5-km fibre link. (**d**) Projected phase noise from the photodiode, rf amplifiers and mixer noise. (**e**) Projected phase noise from the intrinsic thermal noise^28^ of the fibre link.

The measurement results of the absolute timing jitter and the corresponding phase noise spectra are shown in Fig. 3. Note that all phase noise levels are scaled to a 10-GHz carrier frequency for convenient comparison with other state-of-the-art microwave oscillators. Curves **a** and **b** show the repetition rate’s timing jitter/phase noise spectra of free-running and stabilized mode-locked fibre lasers, respectively. Note that the data in 1 Hz–60 kHz and 60 kHz–10 MHz Fourier frequency ranges are measured by the fibre-delay method and the BOC method, respectively. The locking bandwidth used in this study is ~60 kHz, which is also shown in the resonant peak in curve **b**. Using stabilization, the phase noise/timing jitter is suppressed by >30 dB for Fourier frequencies below 1 kHz; this results in the suppression of timing jitter by more than a factor of 100 in the >1-ms time scale. For example, the integrated r.m.s. jitter is suppressed by a factor of 500 from 10 ps (free-running) to only 20 fs (stabilized) over a 1-s time scale. Also, due to the repetition-rate stabilization, sub-femtosecond absolute timing can be maintained by more than 100 times longer (i.e., from 100 μs (free-running) to 10 ms (stabilized)). When computing the fractional frequency instability of the repetition rate from the measured phase noise spectrum^26^, the minimum frequency instability (i.e., Allan deviation) reaches 1.7 × 10^−13^ at a 0.1-s averaging time (see Supplementary Information).

The measured timing jitter/phase noise performance is primarily limited by the degraded RIN floor at 100 MHz (i.e., 2*f*_m_ for synchronous detection) that originated from Rayleigh scattering in a long fibre^27^ for a >20-Hz Fourier frequency range. Curve **c** in Fig. 3 shows the projected phase noise that originated from the measured −133 dB/Hz white RIN, which shows fairly good agreement with the measured result. Note that this limit is higher than the projected phase noise by photodiodes, rf-amplifiers, and mixer (curve **d**) and the fundamental thermal noise limit^28^ of the used fibre-delay (curve **e**). We believe that a longer delay line can improve the phase noise performance because Rayleigh-scattering-induced RIN spectral density at 100 MHz is nearly proportional to the fibre length^27^ (see Supplementary Information).

## Discussion

**Figure 4:**
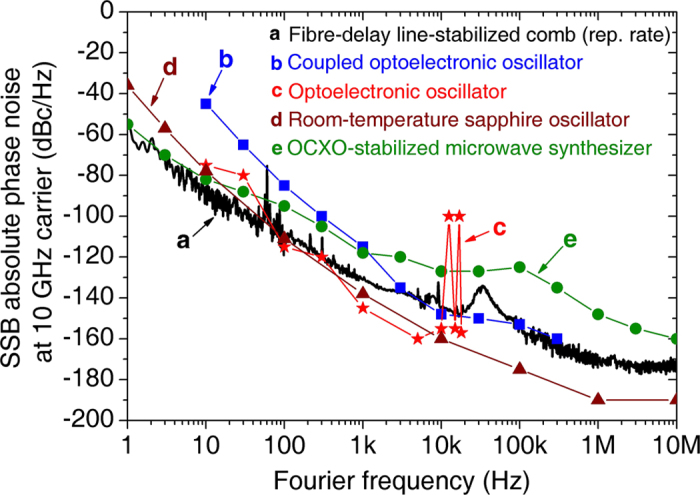
Equivalent phase noise (scaled to a 10-GHz carrier frequency) comparison of the results of this study with those of state-of-the-art room-temperature microwave oscillators. (**a**) Stabilized Er-fibre laser (our work). (**b**) Coupled optoelectronic oscillator (C-OEO)^29^. (**c**) Optoelectronic oscillator (OEO)^29^. (**d**) Room-temperature sapphire oscillator (SLCO-BCS)^30^. (**e**) OCXO-stabilized microwave synthesizer (Keysight Technology, E8257D, opt. UNY)^31^.

We applied a new repetition-rate stabilization method to a mode-locked Er-fibre laser that enables the generation of optical pulse trains with a 980-as (20-fs) absolute r.m.s. timing jitter over a 0.01-s (1-s) time scale using all-fibre photonic technology. As shown in Fig. 4, the equivalent phase noise, which is scaled to a 10-GHz carrier frequency, of the proposed system is already comparable to or even lower than state-of-the-art room-temperature microwave oscillators such as optoelectronic oscillators (OEOs)^29^ and sapphire-loaded cavity oscillators (SLCOs)^30^. Thus, the proposed approach constitutes a new class of photonic signal generators that can be used as a compact, robust, lightweight, and stand-alone attosecond timing source for both optical and microwave domains, which may play an important role in applications such as microwave photonics, optical sampling, time-of-flight ranging and sensing, remote time/timing transfer, and precision synchronization for FEL pump-probe experiments. Conversely, the demonstrated fibre-delay method itself enables simple yet ultrahigh-performance repetition-rate stabilization down to the 10^−13^-level frequency instability within a 0.1-s averaging time. If necessary, longer-term stability can be improved by further stabilizing the associated frequency drift using state-of-the-art quartz clocks or external atomic standards by, for example, inserting a PZT-stretcher as an actuator in the delay arm.

## Methods

### Mode-locked fibre laser

A nonlinear polarization rotation-based, 77-MHz repetition rate, sigma-cavity, passively mode-locked Er-fibre laser is used in this study. This laser operates in the stretched-pulse (i.e., dispersion-managed soliton) regime at +0.002 ps^2^ intra-cavity dispersion with a 15-μm-long PZT (PI, P-840.10)-mounted mirror and an EOM (Thorlabs, EO-PM-NR-C3) for repetition-rate tuning.

### Fibre-delay line interferometer

For the fibre delay, we used a spool of 1.25-km-long, dispersion-compensated fibre, which results in a 2.5-km-long round-trip delay. The fibre was composed of 1.15-km-long standard SMF-28 fibre and 100-m long dispersion-compensating fibre (OFS, LLWBDK). An in-line AOM (Brimrose, AMF-50-1570-2FP) was inserted in the delay arm, which was modulated by a 50-MHz rf source. For the effective interference of the two different wavelength components (i.e., λ_1_ and λ_2_) at the 50:50 coupler, the group delay difference was compensated for using dispersion-compensating fibre (DCF) so that pulse overlap could be achieved for both wavelength components concurrently. For the pulse overlap between the reference arm and the delay arm, the mode-locked laser repetition rate could be tuned up to the inverse delay time (82 kHz) using a 1-inch translation stage inside the laser cavity. However, in case of the out-of-loop fibre-delay interferometer for timing jitter/phase noise measurement, the delay length should be adjusted directly for the pulse-overlap. A 330-ps variable optical delay line (General Photonics, MDL series) was inserted into the delay arm of the out-of-loop fibre interferometer for fine length tuning. The fibre Michelson interferometer was placed in a shielding box with a vibration isolation platform. More information on the shielding effects provided by the shielding box and a vibration isolation platform is provided in the Supplementary Information.

### Repetition-rate stabilization process

A 2-mW optical power of the laser output was used for the repetition-rate stabilization. Two fibre Bragg gratings (FBGs) centred at 1540 nm and 1560 nm with 1-nm FWHM spectral widths were used to select two wavelength sections. Filtered sections were amplified to 14 mW by an EDFA and applied to the Michelson interferometer. A power of 6 mW was applied to each of the delay and reference arms through a 50:50 coupler. Pulses from the round trip in the delay arm experience a 10-dB total loss by fibre pigtailed-AOM insertion loss and splicing loss between the SMF and DCF. The interferometer output was then split into 1540 nm and 1560 nm components by an FBG with a FWHM spectral width of 2 nm. Each spectral component was detected by photodetectors. The incident optical power levels to the photodetectors were 1.7 mW and 0.6 mW for 1560 and 1540 nm, respectively. The obtained 100-MHz rf signals, which were twice the AOM driving frequency, were filtered by rf bandpass filters with a 24-MHz bandwidth to produce >40 dB SNR (measurement resolution bandwidth = 100 kHz). Because a high SNR is important for accurate measurements, cavity-type sharp rf-filters (K&L Microwave, 8LB30-100/T24-O/O) were used to suppress any unnecessary repetition-rate peaks and their harmonics. Finally, 100-MHz rf signals (~−40 dBm) from each spectral component were amplified and frequency-mixed. After the mixer output underwent low-pass filtering, the baseband error signal was obtained. The detected error signal was then split and applied to a PZT and an EOM inside the mode-locked laser cavity through loop filters.

### Frequency noise measurement and calibration

The baseband frequency noise signal from the mixer output is applied to an oscilloscope and a spectrum analyser. The frequency noise detection sensitivity can be expressed as 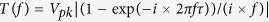 [*V*/Hz], where *V*_*pk*_ is the low-pass filtered mixer output voltage amplitude of interference pattern, *f* is the Fourier frequency, and τ is the round-trip delay time between the two arms in the interferometer^21^. When 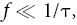 the sensitivity is constant and equal to 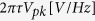. For calibration, the laser PZT input voltage is modulated, and the measured mixer output peak-to-peak voltage is equal to 2*V*_*pk*_. The repetition-rate frequency noise is measured at the difference frequency of 2.5 THz between the used two spectral components (i.e., 1540 nm and 1560 nm). We convert the frequency noise spectral density into the phase noise and scale the 2.5-THz carrier down to 10-GHz for convenient comparison with other microwave oscillators.

## Additional Information

**How to cite this article**: Jung, K. and Kim, J. All-fibre photonic signal generator for attosecond timing and ultralow-noise microwave. *Sci. Rep.* **5**, 16250; doi: 10.1038/srep16250 (2015).

## Supplementary Material

Supplementary Information
