# Supplementary material for: All-fibre photonic signal generator for attosecond timing and ultralow-noise microwave
Source: Sci Rep. 2015 Nov 4;5:16250. doi: 10.1038/srep16250 (PMC4632029; doi:10.1038/srep16250)
Supplement: Supplementary Information [file srep16250-s1.pdf]

Supplementary Information –

All-fibre photonic signal generator for attosecond timing and ultralow-noise microwave

Kwangyun Jung & Jungwon Kim*

School of Mechanical and Aerospace Engineering, Korea Advanced Institute of Science and Technology (KAIST), Daejeon 305-701, Korea

**e-mail: jungwon.kim@kaist.ac.kr*

1. Out-of-loop absolute phase noise/timing jitter measurement methods

For the out-of-loop assessment of absolute phase noise in repetition-rate, two different methods are used in a complementary way. Figure S1 shows the schematic of measurement setup. First, we built another fibre-delay line-based interferometer setup and used it for the out-of-loop phase detector[S1] (Fig. S1a). 2-mW optical power from the mode-locked fibre laser is applied to the out-of-loop fibre-interferometer system. The baseband frequency noise signal from the mixer output is measured and converted to the equivalent phase noise spectral density. Note that frequency drift of the two independent interferometer systems is so low that slow feedback loop is not required during the measurement time up to 20 min. As the frequency noise detection sensitivity can be expressed as

$$T(f) = V_{pk}\left|(1-\exp(-i\times 2\pi f\tau))/(i\times f)\right| \ [V/\mathrm{Hz}], \qquad \text{(Eq. 1)}$$

where V_{pk} is the low-pass filtered mixer output voltage amplitude of interference pattern, f is Fourier frequency, and τ is the round trip delay time between the two arms in the interferometer [S2], the measurement sensitivity is degraded for Fourier frequency above the first null at $1/\tau$. As shown in Eq. 1, because null points in the interferometer sensitivity happen at every integer multiple of the inverse delay time (e.g., 82 kHz and its harmonics in this work)[S3,S4], the delay-line measurement shows many spikes in every integer multiple of the inverse delay time, which is not real laser noise but a measurement artefact (see the measurement result in Fig. S2). As a result, we use the measured data by the out-of-loop fibre-delay line only for <60 kHz Fourier frequency.

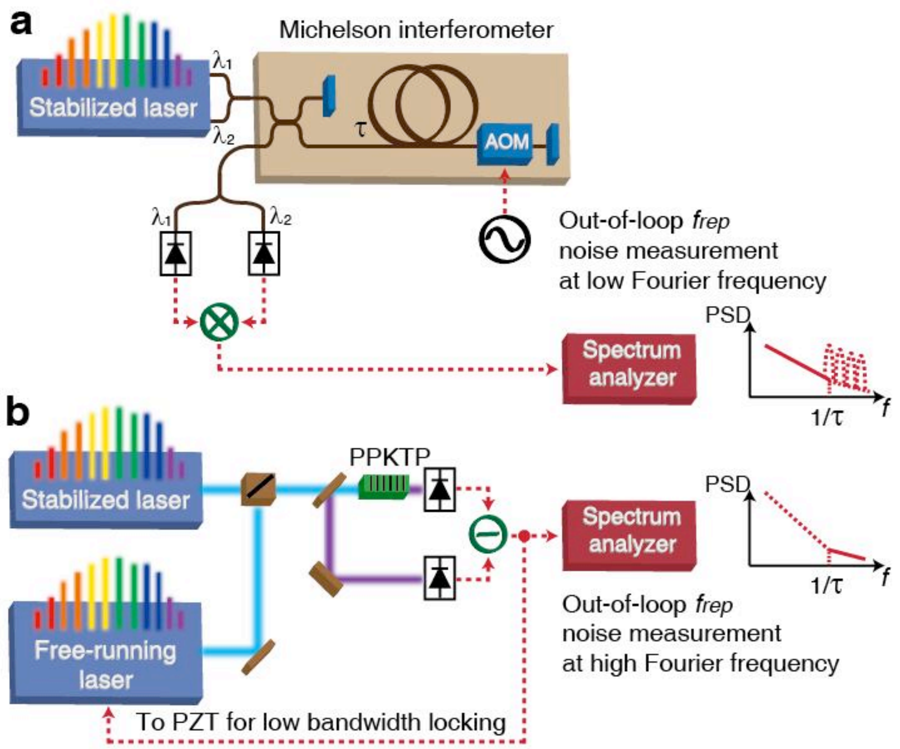

Figure S1 | Schematic of out-of-loop absolute timing jitter/repetition-rate phase noise spectrum measurement. a Using the out-of-loop fibre-delay line-based measurement. **b** Using the balanced optical cross-correlation (BOC) measurement. Here, stabilized laser means that repetition-rate is already stabilized to the in-loop fibre-delay line (not shown in this figure). AOM, acousto-optic modulator; PPKTP, periodically poled $KTiOPO_4$; PZT, piezoelectric transducer.

For above 60 kHz Fourier frequency, we use the well-established balanced optical cross-correlation (BOC) method[S5]. The BOC method requires two independent mode-locked lasers for timing comparison. Therefore, we additionally built an independent free-running mode-locked Er-fibre laser, which was designed in a similar way with the stabilized mode-locked laser. Above ~60 kHz Fourier frequency (locking bandwidth of the fibre-delay line-based stabilization), the stabilized laser and the free-running laser will have a similar level of timing jitter/phase noise spectral density. Therefore, the BOC measurement between the stabilized laser and the free-running laser sets the upper limit in timing jitter spectral density of the stabilized laser above ~60 kHz Fourier frequency.

In order to crosscheck the validity of fibre-delay line method for measuring timing jitter and repetition-rate phase noise, we compare the delay line measurement result with the BOC result. Figure S2 shows the timing jitter/repetition-rate phase noise spectra of free-running mode-locked Er-fibre lasers measured by two different methods. The delay line result for the higher-jitter laser and the BOC result show a good agreement below 82 kHz (1/τ frequency for 2.5-km delay), which confirms the validity of fibre-delay line method for measuring timing jitter and repetition-rate phase noise spectra within 1/τ frequency. As discussed in page 1 of Supplementary Information, the delay-line measurement results show spikes at multiple of the inverse delay time (*k*/τ, where *k*=1,2,3…), which is the measurement artefact caused by null points in the *T*(*f*) transfer function (Eq. 1).

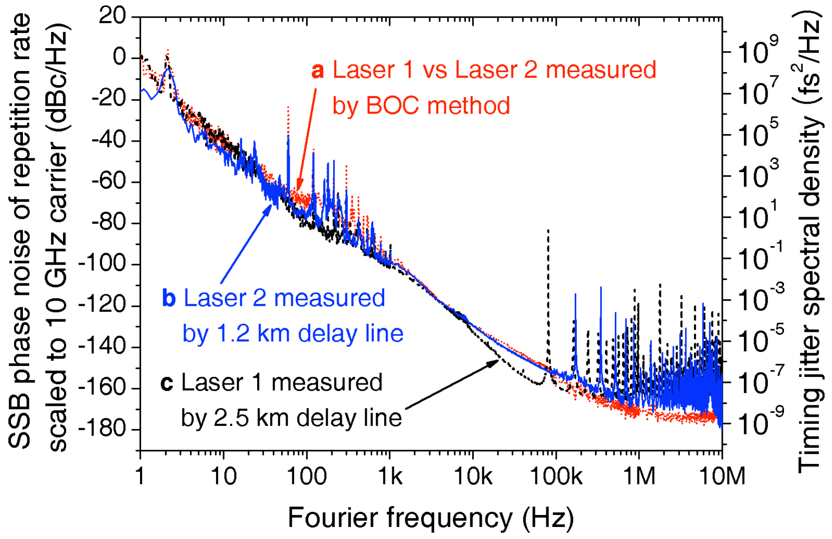

Figure S2 | Comparison of timing jitter measurement results of free-running mode-locked Er-fibre lasers.

2. Scalability in phase noise/timing jitter spectra

Wavelength spacing: In order to improve the phase detection sensitivity and therefore the achievable stabilization performance, the wavelength spacing (λ_1 - λ_2) can be

increased. As the detection sensitivity of repetition-rate frequency noise is proportional to the frequency difference between the used spectral ranges, $(m\text{-}n)f_{rep}$, for the best performance, it will be desirable to use as wide frequency difference as possible, which is allowed by the spectrum of the used mode-locked laser.

Scaling by delay time: In addition, since the absolute frequency noise of the comb modes is amplified by the delay time, longer delay line makes higher measurement sensitivity below the Fourier frequency of inverse delay time (1/ τ): for $f \ll 1/\tau$, the sensitivity is $2\pi\tau V_{pk}[V/Hz]$, where V_{pk} is the low-pass filtered mixer output voltage amplitude of interference pattern and τ is the round trip delay time between the two arms in the interferometer. However, the interferometer transfer function has null points at every integer multiple of the inverse delay time (as shown in Eq. 1). As a result, the first null point at 1/τ Fourier frequency sets the limitation in the achievable locking bandwidth. There should be a trade-off between the achievable sensitivity (which is proportional to τ) and the achievable bandwidth (which is proportional to 1/τ), depending on the intrinsic repetition-rate phase noise level of the used free-running mode-locked laser. Despite the restriction in the achievable locking bandwidth, one important advantage of using long fibre-delay line as a means for repetition-rate stabilization is that it is nearly laser repetition-rate independent, due to much smaller 1/τ frequency (e.g., ~100 kHz for 2-km long fibre delay) compared to the free spectral range of typical ultrahigh-Q cavities. By slightly tuning the repetition-rate of the laser and/or tuning the length of fibre-delay line, one can easily find the right locking point regardless of the intrinsic repetition-rate of the used mode-locked laser.

Impact of scattering-limited RIN: Although longer fibre length makes more sensitive detection sensitivity, it also increases scattering-limited RIN. Therefore, the scattering issue should be also considered. As shown in Fig. 3 of main paper, in our experiment, the stabilized timing jitter/phase noise performance for >20-Hz Fourier frequency range

with shielding box and vibration isolation was mostly limited by the Rayleigh scattering-limited RIN floor at 100 MHz (which is $2f_m$ for synchronous detection). As experimentally studied in ref. S6, Rayleigh scattering-induced RIN spectral density (with 1/Hz unit) at >1-MHz frequency is almost proportional to the fibre length (L). Our system also had the same scaling factor as the synchronous detection is performed at 100 MHz. As the RIN-projected phase noise spectral density (with rad^2/Hz unit) is proportional to $1/L^2$ below the $1/\tau$ Fourier frequency, it means that longer delay line can improve the stabilized repetition-rate phase noise performance when limited by the Rayleigh scattering-induced RIN.

Impact of power-dependent RIN: It is also worthwhile to note that optical power dependent RIN by the long fibre also influences the stabilized phase noise/timing jitter spectra. When the input power to the fibre link increases, the Rayleigh scattering-induced RIN gets lower at >10-kHz[S7]. However, when the power level exceeds the threshold of stimulated Brillouin scattering, the RIN increases rapidly[S7]. As a result, in our experiment, 6 mW input power to the delay arm enabled the lowest RIN and the best stabilization performance, which is also consistent with the results shown in ref. S7.

Projected performance when a lower-jitter mode-locked laser is used: The 2.5-km delay length in the main paper is the optimized length for minimizing the total integrated jitter when a mode-locked laser with ~840-as high-frequency [10 kHz – 10 MHz] jitter is used. If a mode-locked laser with lower intrinsic timing jitter is used, we can reduce the necessary locking bandwidth and increase the delay length, which in turn will improve the stabilized repetition-rate phase noise performance. For example, if a recently demonstrated Er:Yb-glass laser with a 16-as jitter [10 kHz – 5 MHz][S8] is employed, the projected absolute timing jitter is 360-as [100 Hz – 10 MHz] by using a 25-km-long fibre delay line (with 10 dB lower phase noise within the locking bandwidth of 6 kHz).

3. Impact of shielding on repetition-rate phase noise/timing jitter

The Michelson interferometer (with a footprint of 30 cm by 30 cm; Fig. S3a) is protected by a double-walled shielding box (Fig. S3b) and a passive vibration isolation platform (Fig. S3c) for the best performance. Figures S3d and S3e show the impact of shielding on phase noise spectra and frequency instabilities, respectively. The outer wall is a 5-mm thick aluminium case, and 2-mm thick sound insulation material is attached on it. The inner wall is a 5-mm thick Delrin (thermal insulation) case, and 50-mm thick sound-absorbing material is attached inside the Delrin box. The shielding box is put on a passive vibration isolation platform (Minus-k Technology, 50BM-10). Temperature of the shielding box is not actively controlled. We measure the out-of-loop repetition-rate phase noise of the stabilized fibre laser without shielding and vibration isolation platform (Fig. S3a), with shielding but no vibration isolation platform (Fig. S3b), and with both shielding and vibration isolation platform (Fig. S3c). The measured phase noise level is almost identical for >1 kHz Fourier frequency regardless of shielding or vibration isolation. However, the shielding box effectively suppresses phase noise bumps induced by acoustic noise in the 100 Hz - 1 kHz Fourier frequency range by ~10 dB. When the shielding box is not used, the integrated timing jitter is 3.2 fs [100 Hz – 1 kHz], whereas the jitter is reduced to 0.75 fs [100 Hz – 1 kHz] when the shielding box is used. This measurement result suggests that the timing jitter originated from the acoustic noise is 3.1 fs [100 Hz – 1 kHz] in our experimental environment. In addition, the shielding box prevents rapid phase diffusion induced by thermal drift below 1 Hz. The vibration isolation platform further improves phase noise below 30 Hz Fourier frequency.

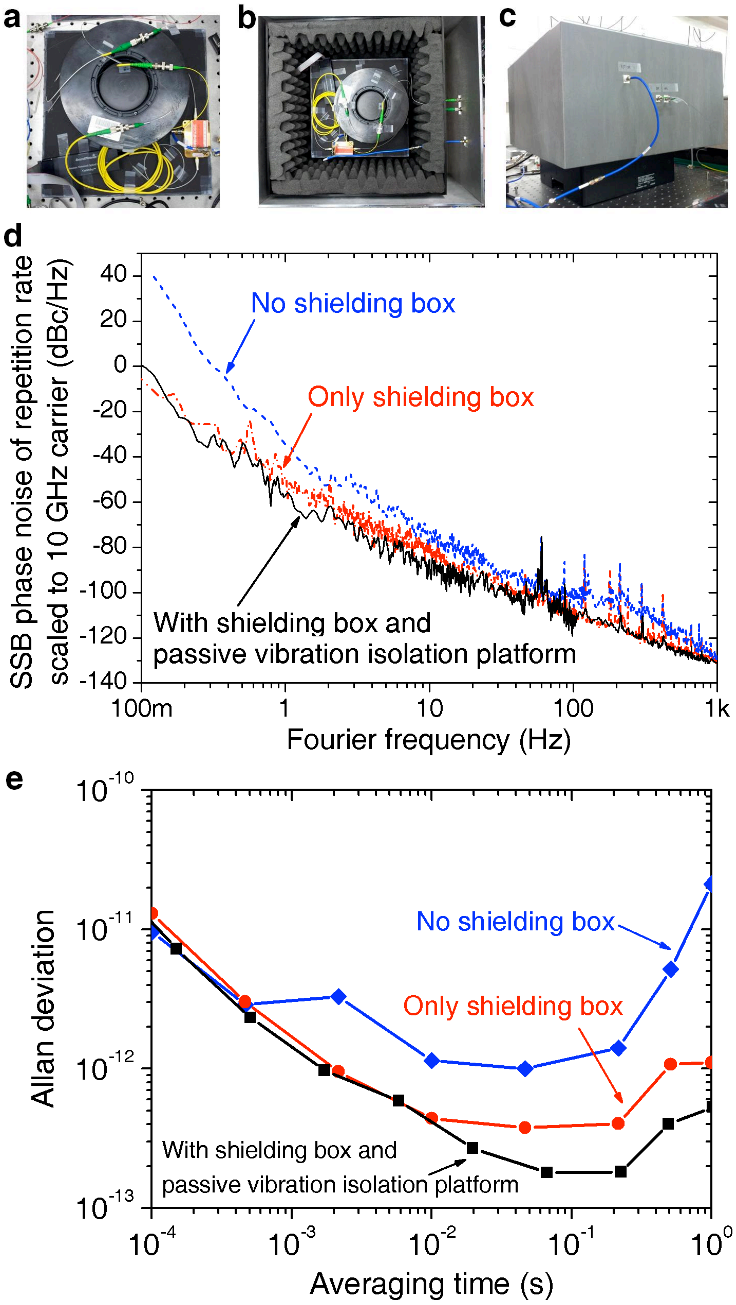

Figure S3 | Impact of shielding on repetition-rate phase noise/timing jitter. a Michelson interferometer without shielding or vibration isolation platform. **b** Shielding case. **c** Vibration isolation platform. **d** Measured timing jitter/repetition-rate phase noise of the stabilized Er-fibre laser from 0.1 Hz to 1 kHz Fourier frequency. **e** Fractional frequency instability of repetition-rate in terms of Allan deviation.

Figure S3e shows the fractional frequency instability in repetition-rate for each case, calculated from the measured phase noise spectra[S9]. With shielding case and vibration isolation, the minimum frequency instability reaches 1.7 x 10^{-13} at 0.1-s

averaging time, and it rapidly increases to 5.3×10^{-13} at 1-s averaging time due to thermal drift. Note that the fractional frequency instability of the repetition rate follows the fractional optical path length stability of the fibre spool (δL/L). As the temperature coefficient of the fractional optical path length is ~10^{-5}/K for typical fibre[S10], the temperature change can be very low in short-term time scale (e.g., <1 s) if a sufficient thermal isolation is used. Our result of 5.3×10^{-13} Allan deviation at 1-s averaging time means that temperature change is below 70 nK in 1-s time scale. With the shielding case and vibration isolation platform, the measured linear frequency drift is 2.5 Hz/s at 2.5 THz. Note that even without vibration isolation platform, the frequency instability can reach 3.9×10^{-13} at 0.1-s averaging time. When the shielding case is further removed, the minimum frequency instability still reaches 1.0×10^{-12} at 0.05-s averaging time, which underlines the robustness of the demonstrated all-fibre-delay line-based stabilization method.

4. Discussions

Subfemtosecond-jitter, ultralow phase noise microwave generation. The demonstrated technique constitutes for a stand-alone photonic signal generator that can generate an optical pulse train with sub-fs timing jitter in 0.01-s time scale [100 Hz – 10 MHz]. By proper optical-to-electronic (O-E) conversion of optical pulse trains, ultralow phase noise microwave signals at the harmonic of fundamental repetition-rate can be generated as well. For example, when we employ a fibre-loop optical-microwave phase detector (FLOM-PD) and a dielectric resonator oscillator (DRO) to generate 10-GHz microwave signal as shown in ref. S11, the projected integrated jitter is 1.1 fs from 100 Hz to 10 MHz Fourier frequency, which shows that 490-as excess jitter is added by the O-E conversion process. If high-repetition rate optical pulse trains and a state-of-the-art high-power and high-linearity photodiode are employed as ref. S12, the additional jitter in O-E conversion can be further suppressed to 160-as. Even a typical phase noise floor of -145 dBc/Hz for O-E conversion (by standard high-speed photodiodes and fibre

lasers) is assumed[S13], the integrated jitter is 4.1 fs with the contribution from O-E conversion of 4.0 fs [100 Hz – 10 MHz], which is still far better than most of state-of-the-art room-temperature microwave generators. Therefore, this simple all-fibre-optic signal generator can be a compact, stand-alone source for applications that require both ultralow-noise optical pulse trains and microwaves, such as master oscillators for free-electron laser (FEL) timing and synchronization[S14], master sources for photonics radars[S15] and time-of-flight-based ranging[S16], and sampling clocks for photonic analogue-to-digital converters[S17] and down-conversion receivers[S18] to name only a few.

Implementation at other wavelength ranges. Although the demonstration was performed at 1550-nm telecommunication wavelength in this work, we believe that the method can be extended to other wavelengths as well, including 1-μm (Yb-comb) and 800-nm (Ti:sapphire comb) ranges. In our experiments, we found that dispersion compensation and pulsewidth are not critical factors in obtaining high SNR. As long as the pulses overlap well with proper group delay adjustment for the two wavelength components, high SNR could be obtained, regardless of the pulsewidth after long fibre. Therefore, we believe that our method may be applicable to other wavelengths as well. The important issue is to independently adjust the group delays for the two wavelength components for effective interference, which can be performed by wavelength split, independent delay length tuning, and recombination.

References

S1. Cranch, G. A. Frequency noise reduction in erbium-doped fiber distributed-feedback lasers by electronic feedback. *Opt. Lett.* **27**, 1114-1116 (2002).

S2. Sheard, B. S., Gray, M. B. & McClelland, D. E. High-bandwidth laser frequency stabilization to a fiber-optic delay line. *Appl. Optics* **45**, 8491-8499 (2006).

S3. Rubiola, E., Salik, E., Huang, S., Yu, N. & Maleki, L. Photonic-delay technique for phase-noise measurement of microwave oscillators. *J. Opt. Soc. Am. B* **22**, 987-997 (2005).

S4. Eliyahu, D., Seidel, D. & Maleki, L. Phase noise of a high performance OEO and an ultra low noise floor cross-correlation microwave photonic homodyne system. in *Proceedings of the IEEE International Frequency Control Symposium*, 811-814 (2008).

S5. Jung, K. & Kim, J. Characterization of timing jitter spectra in free-running mode-locked lasers with 340 dB dynamic range over 10 decades of Fourier frequency. *Opt. Lett.* **40**, 316-319 (2015).

S6. Cahill, J. P., Okusaga, O., Zhou, W., Menyuk, C. R. & Carter, G. M. Superlinear growth of Rayleigh scattering-induced intensity noise in single-mode fibers. *Opt. Express* **23**, 6400-6407 (2015).

S7. Okusaga, O. *et al.* Optical scattering induced noise in rf-photonic systems. in *Proceedings of the IEEE International Frequency Control Symposium & European Frequency and Time Forum*, 994-999 (2011).

S8. Hou, D. *et al.* Attosecond timing jitter characterization of mode-locked lasers using optical heterodyne techniques. in *Proceedings of Conference on Lasers and Electro-Optics*, SF1L.5 (2015).

S9. Riley, W. J. *Handbook of Frequency Stability Analysis* (NIST Special Publication 1065, 2008).

S10. Chen, Y. T. Use of single-mode optical fiber in the stabilization of laser frequency. *Appl. Optics* **28**, 2017-2021 (1989).

S11. Jung, K., Shin, J. & Kim, J. Ultralow phase noise microwave generation from mode-locked Er-fiber lasers with subfemtosecond integrated timing jitter. *IEEE Photon. J.* **5**, 5500906 (2013).

S12. Baynes, F. N. *et al*. Attosecond timing in optical-to-electrical conversion. *Optica* **2**, 141-146 (2015).

S13. Quinlan, F. *et al*. Ultralow phase noise microwave generation with an Er:fiber-based optical frequency divider. *Opt. Lett*. **36**, 3260-3262 (2011).

S14. Kim, J., Cox, J. A., Chen, J. & Kärtner, F. X. Drift-free femtosecond timing synchronization of remote optical and microwave sources. *Nat. Photon*. **2**, 733-736 (2008).

S15. Ghelfi, P. *et al*. A fully photonics-based coherent radar system. *Nature* **507**, 341-345 (2014).

S16. Lee, J., Kim, Y.-J., Lee, K., Lee, S. & Kim, S.-W. Time-of-flight measurement with femtosecond light pulses. *Nat. Photon*. **4**, 716-720 (2010).

S17. Valley, G. C. Photonic analog-to-digital converters. *Opt. Express* **15**, 1955-1982 (2007).

S18. Kim, J., Park, M. J., Perrott, M. H. & Kärtner, F. X. Photonic subsampling analog-to-digital conversion of microwave signals at 40-GHz with higher than 7-ENOB resolution. *Opt. Express* **16**, 16509-16515 (2008).
